# 

**Published:** 1915-08-28

**Authors:** 


### CONTENTS.

| | PAGE |
|---|---|
| **The Finances of the Voluntary War Funds** ... | 443 |
| **The Public and Doctors for the Army** ... | 444 |
| **Hospital and Institutional News** ... ... ... | 445 |
| Death of Professor Ehrlich—Medical Specialism at the Front—A Great Canadian Hospital at Orpington—Mr. Ian Malcolm, M.P.—New Convalescent Homes—Dr. Robert Sinclair, of Dundee—Offenders in High Places—The Difficulties of Personal Identification—Red Cross Work in Aberdeenshire—Gifts and Service in War-Time—Results of Treatment in German Hospitals—D.S.O. for an Ambulance Worker—Expeditious Red Cross Service—Repairs and Maintenance—Fever Hospital Humours—Legatees and Legacies—The Homœopathic Hospital and the Wounded—The Three Years' System of Treasurerships—The Shortage of Cube Sugar—Dr. Walker, of Peterborough—The New Secretary of the Stockport Infirmary—Measles and the Public—The Louse and Its Relation to Disease—A Surgeon's Experience at the Front—The L.G.B., the Guardians, and Economy—This Week's Drug Market. | |
| **On Hemiplegia in Childhood** ... ... ... | 451 |
| **"The Uric Acid Fetish"** ... ... ... ... | 452 |
| The Decline of its Flesh-creeping Power. | |
| **Some Results of War Time** ... ... ... | 453 |
| Shortage in X-Ray Operators. | |
| The War Pinch on the Huns. | |
| **Research and Remedies** ... ... ... ... | 454 |
| Acetone and Vomiting. | |
| The Luetin Test for Syphilis. | |
| Diet in Advanced Heart Disease. | |
| **In a General Hospital in France** ... ... ... | 455 |
| Miss Lena Ashwell's Concert Parties. | |
| **The Alton Hospital for Cripples**... ... ... | 456 |
| Treatment of Tuberculous Bone Disease. | |
| **The Revised Uniform System of Accounts** ... | 457 |
| The Three Funds Satisfied. | |
| **The Leicester Saturday Hospital Society** ... | 458 |
| War Effects on Sickness. | |
| **Round the World** ... ... ... ... ... | 459 |
| Fellow-Workers and the Roll of Honour. | |
| **The New Physiological Buildings at Cardiff** ... | 460 |
| Sir William Osler's Wise Counsel. | |
| **The Editor's Letter-Box** ... ... ... ... | 460 |
| War Service for Resident Medical Officers. | |
| **Personalia** ... ... ... ... ... ... ... | 460 |
| **Buildings Contemplated and in Progress** ... | iv |
| **The Book Market** ... ... ... ... ... ... | iv |
| **Institutional Needs** ... ... ... ... ... ... | iv |
| **Latest Installations in Hospital Equipment** ... | iv |
| What Others are Doing. | |
| **The Institutional Worker** ... ... | (Suppl.) |
| **A Hospital's Publicity Department.** | |
| Some Hints, Ideas, and Debateable Points: By a Secretary. | |
| Question for August. | |
| Questions Invited. | |
| Enquiries and Answers. | |
| The Sick and In Need. | |
| Practical Matters. | |
| Employment and Training. | |
| The Housekeeper's Department. | |
| Approved Home. | |

